# Smoking-induced chromosomal segregation anomalies identified by FISH analysis of sperm

**DOI:** 10.1186/s13039-014-0058-7

**Published:** 2014-09-12

**Authors:** Ciro Silveira Pereira, Maria Silvina Juchniuk de Vozzi, Silvio Avelino dos Santos, Maria Aparecida C Vasconcelos, Cláudia CP de Paz, Jeremy A Squire, Lucia Martelli

**Affiliations:** Department of Genetics, Ribeirao Preto Medical School, University of São Paulo, 3900 Bandeirantes Avenue, Zip code 14049-900 Ribeirao Preto, SP Brazil; Department of Gynecology and Obstetrics, Ribeirao Preto Medical School, University of São Paulo, 3900 Bandeirantes Avenue, Ribeirão Preto, SP Brazil; Department of Pathology and Molecular Medicine, Queen’s University, 88 Stuart Street, Kingston, ON Canada

**Keywords:** Tobacco consumption, Sperm, FISH, Meiosis, Male gametogenesis, Disomy, Male infertility, Aneuploidy, Non-disjunction

## Abstract

**Background:**

Numerical chromosome aberrations in gametes are directly related to infertility and aneuploid embryos. Previous studies have shown that toxic substances from cigarette smoke induce structural and numerical chromosomal aberrations in vitro and could potentially increase levels of aneusomy in sperm. Moreover, increased levels of aneusomy in sperm are correlated with low implantation rates, spontaneous abortions and fetal losses. Studies of chromosome 3 in sperm suggest it may be more prone to segregation anomalies than other autosomes, but there has been no systematic investigation of the incidence of disomy for chromosome 3 in sperm derived from donor male smokers. The objective of this study was to use FISH to evaluate the influence of smoking on the levels of disomy for chromosomes X and Y, and to determine whether disomy levels for chromosome 3 were elevated in sperm derived from male smokers.

**Results:**

FISH analysis was used to evaluate the frequency of disomies of chromosomes 3, X, and Y in sperm of 10 smokers, compared to a control group of 7 non-smoking fertile men. All the subjects presented a normal somatic karyotype. There was a significant increase in the overall frequency of disomies in sperm derived from the smoking group (P< 0.0001). When each chromosome pair was analyzed individually, disomy of chromosome 3 in smokers was found to be more than twice that observed in the matched non-smoker control group. In addition we observed a higher frequencies of disomy of the X and Y chromosomes, indicating elevated levels of diploidy in the sperm from the smoking group.

**Conclusions:**

In this study we have shown that chromosome 3 may be susceptible to smoking-related segregation anomalies. Our results also suggest that errors can occur in both meiosis I and II, confirming the emerging literature that the male meiotic process may generally be affected by the genotoxic damage from tobacco use. Collectively, these findings provide additional evidence for enhancing tobacco control measures, and suggest that FISH analysis of chromosome 3 in sperm may be useful for monitoring smoking–induced segregation damage as part of the evaluation of infertile males.

## Background

Numerical chromosome abnormalities are directly related to infertility. Approximately 35% of spontaneous abortions in the first trimester are caused by some chromosomal anomaly [1]. Errors in paternal meiosis are responsible for about 5-10% of autosomal aneuploidies; for nearly 50% of 47,XXY; for 70% of 45,X; and for all of the 46,XYY sex chromosome anomalies [2].

Advanced maternal age still remains as one of the few well-established risk factors. Other potential contributors to aneuploidy include alcoholism, occupational exposure to pesticides, and smoking [3]. The World Health Organization [4] estimates that about one-third of the male population smokes. Furthermore, cigarettes contain more than 400 toxic substances and more than 4,000 different chemicals with unknown physiological consequences. Exposure to tobacco smoke has been experimentally shown to induce cellular DNA damage and numerical and structural chromosomal anomalies in mammalian and prokaryotic models, including in vitro and in vivo systems [5–7].

Honein and colleagues [8] observed a positive association between maternal smoking and birth defects, including hydrocephaly, microcephaly and oral clefts. Recently, significant associations with maternal smoking were also found for cardiovascular/heart defects, musculoskeletal defects, limb reduction and other birth defects [9].

Furthermore, in vitro studies confirmed that amniocytes obtained from pregnant women and cultured in medium containing nicotine showed high frequencies of structural and numerical chromosomal aberrations, involving chromosomes 21, 22, 8, 15, and 20 [10].

In males, it was verified that cotitine, a metabolite from nicotine, is able to cross the hematotesticular barrier, but tissue effects remain unknown [11,12]. There are previous reports of an increase in disomy associated with cigarette smoking (reviewed in Templado [13]), suggesting that molecular cytogenetic approaches are likely to be informative.

Interphase fluorescence in situ hybridization (FISH) has been used with great success to enumerate chromosomal aneuploidies in germ and sperm cells. This technique makes possible a faster and simpler analysis of numerical abnormalities in a high number of cells in a short time period [14,15] and was previously used to evaluate correlations between aneusomies in sperm and exogenous or endogenous factors like fungicides [16], pesticides [17,18], chemotherapy [19], age [20], seminal parameters, reduced sperm count [21,22] and clinical implications in Assisted Reproduction [23,24].

Trisomy 21 (Down syndrome), monosomy X (Ullrich-Turner syndrome) 47,XXY (Klinefelter) and triple X are the most common numerical chromosomal syndromes in newborns [25,26]. Numerical chromosome abnormalities involving chromosomes 13, 16, 21, 22, and sex chromosomes are common causes of spontaneous abortions [27]. However, there are few interphase FISH studies evaluating the meiotic segregation of chromosome 3 in germ cells. The accepted consensus is that large metacentric and submetacentric chromosomes are subject to less meiotic errors due to their large synaptonemal complex, and the relative increase in the number of crossing over events as the bivalents pair. There is only one previous study of chromosome 3 in normosomic controls in which a higher frequency of disomy was observed in comparison to other autosomal chromosomes [28]. This observation suggests that this chromosome may be more prone to segregation anomalies, and may thus also be a more informative overall monitor of smoking-induced meiotic damage. Similarly interphase analysis of the sex chromosomes can distinguish the stage of meiosis that could be more susceptible to smoking-related segregation anomalies. Therefore analysis of sex chromosomes was included as the XY FISH probe can differentiate between abnormalities mostly occurring during meiosis I (responsible for XY disomy) from those taking place in meiosis II (causing XX and YY disomies) [2,29]. Is noteworthy that some of these XX and YY disomies can take place previously in meiosis I by premature separation of sisters chromatids or all of the disomies can be generated by pre-meiotic events [29,30].

The goal of this study was to determine whether chromosome 3 was subject to meiotic segregation anomalies in sperm derived from male smokers. The approach taken was to investigate the frequency of disomies and diploidies in sperm cells of smokers and in suitably matched control donors, to determine whether there was an association between numerical chromosomal abnormalities and smoking. We selected the most informative combination of DNA probes from chromosomes 3, X, and Y to monitor the extent and type of meiotic segregation error that could be associated with smoking.

## Results

Chromosome analysis by GTG banding revealed a 46,XY karyotype for all controls and smokers included in the cohort. There was no difference in the mean age between the two groups which were 33 ± 2.9 years old (mean ± SD) for controls and 33 ± 3.1 for the smoking group.

Table 1 shows comparisons of the seminal parameters for the two groups, smokers and nonsmokers. A significant difference in morphology (*P* < 0.036) was observed, and the smoking group showed a diminished lower number of morphological normal cells (7.7 ± 4.5) compared with the control group (13.1 ± 5.1).

**Table 1:** **Seminal parameters of the controls and smokers**

	**Controls**								**Smokers**										
	**C1**	**C2**	**C3**	**C4**	**C5**	**C6**	**C7**	**Mean ± SD**	**P1**	**P2**	**P3**	**P4**	**P5**	**P6**	**P7**	**P8**	**P9**	**P10**	**Mean ± SD**
Volume (ml)	2.0	3.9	3	1.2	4.5	3.2	4.1	3.1 ± 1	2.2	2.4	0.7	3	3.2	5	1.7	2.2	2	1.5	2.4 ± 1
Concentration × 10^6^ spz/ml	79.5	50	35	100	39	96.5	69	67 ± 26	54.5	75.6	66.5	85	26.5	74.5	19	21.5	119	29	57.0 ± 33
Total sperm count × 10^6^	159	195	102	120	175.5	308.8	282.8	191.9 ± 78	119	181.44	46.55	255	84.8	372.5	31.45	47.3	238	43.5	142.0 ± 115
PR	75	44	32	39	27	43	35	42.1 ± 15	18	71	24	32	23	29	33	15	27	12	28.4 ± 16
NP	17	24	43	37	42	36	35	33.4 ± 9	48	24	42	39	47	41	39	37	37	43	39.7 ± 6
IM	8	32	25	24	31	21	30	24.4 ± 8	34	5	34	29	30	30	28	48	36	45	31.9 ± 11
Vitality %	94	83	87	86	89	93	87	88.4 ± 4	90	98	93	93	90	90	92	89	80	75	89.0 ± 7
Morphology %	21	9	8	13	8	17	16	13.1 ± 5	4	3	10	15	13	12	8	5	4	3	7.7 ± 4

**PR:** Sperm with progressive motility.

**NP:** Sperm with non-progressive motility.

**IM:** Sperm immotile.

**spz/ml:** spermatozoa per ml.

**SD:** standard deviation.

**Figure 1:**
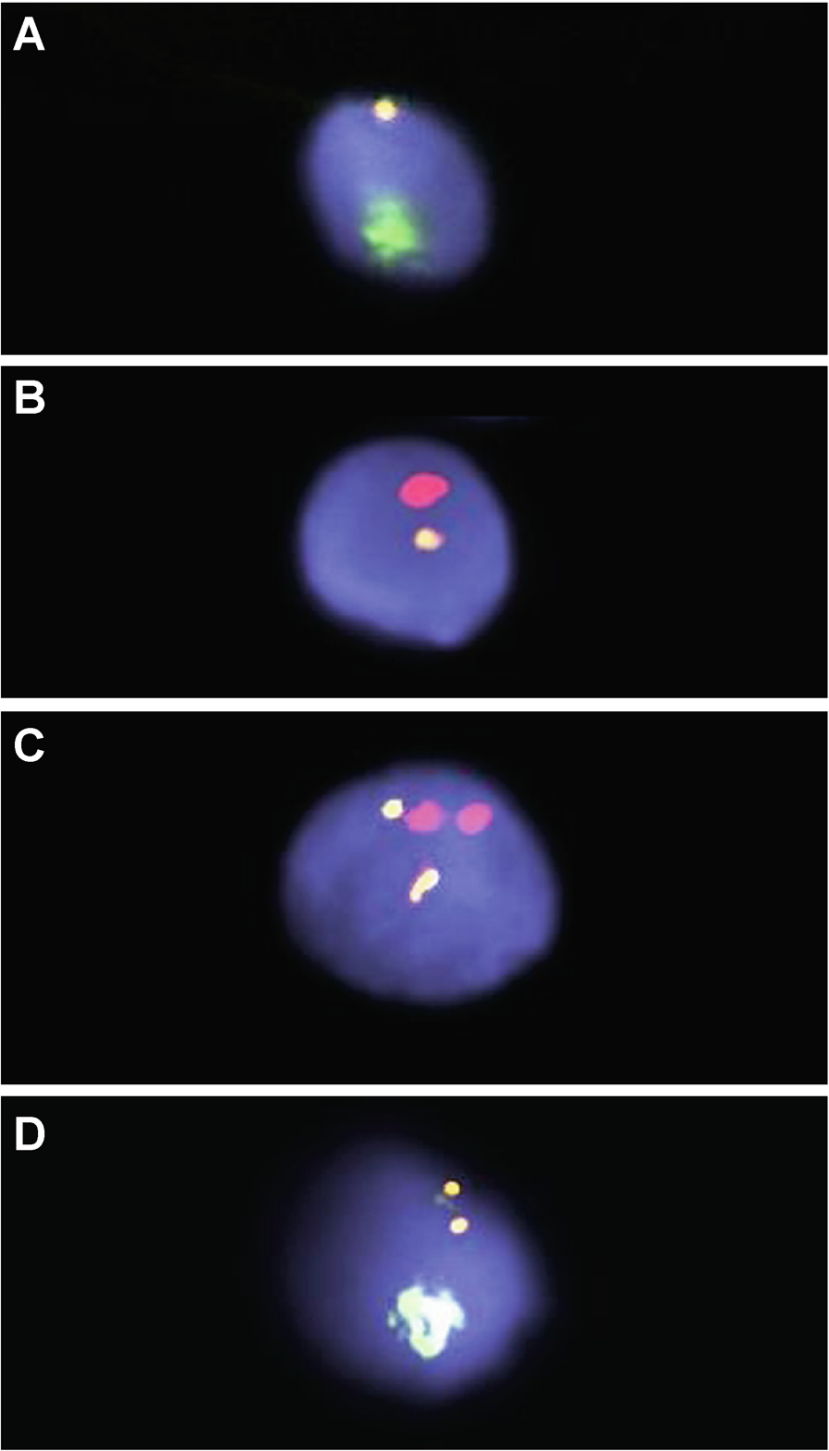
**FISH analysis of sperm. A**: FISH analysis of sperm showing one chromosome Y (Yqh spectrum green) and chromosome 3 (D3Z1 yellow); **B**: FISH analysis of sperm showing one chromosome X (DXZ1 spectrum red) and chromosome 3 (D3Z1 yellow); **C**: FISH analysis of diploid sperm showing two signals for chromosome X (red) and two signals for chromosome 3 (yellow): **D**: FISH analysis of disomy of chromosome 3 (yellow) and chromosome Y (Yqh spectrum green).

A total of 14,014 sperm cells from nonsmokers (control group) and 20,197 from smokers (study group) were evaluated for aneusomy (Figure 1) of the chromosomes 3, X, and Y. The data are shown in Table 2.

**Table 2:** **Percentage (and absolute count) of disomies and diploidies of chromosomes 3, X and Y in controls and smokers**

	**Disomies**							**Diploidies**			
	**X3**	**Y3**	**XY3**	**XX3**	**YY3**	**X33**	**Y33**	**XY33**	**XX33**	**YY33**	**Total**
**Controls**											
**C1**	50.57 (1,024)	48.54 (983)	0.20 (4)	0.15 (3)	0.15 (3)	0.05 (1)	0.10 (2)	0.20 (4)	0 (0)	0.05 (1)	100 (2,025)
**C2**	44.30 (886)	54.25 (1,085)	0.65 (13)	0.25 (5)	0.20 (4)	0 (0)	0.05 (1)	0.25 (5)	0.05 (1)	0.00 (0)	100 (2,000)
**C3**	49.61 (957)	49.30 (951)	0.16 (3)	0.05 (1)	0.10 (2)	0.05 (1)	0.16 (3)	0.41 (8)	0.10 (2)	0.05 (1)	100 (1,929)
**C4**	50.57 (1,015)	49.08 (985)	0.15 (3)	0.05 (1)	0.05 (1)	0.05 (1)	0 (0)	0.05 (1)	0 (0)	0 (0)	100 (2,007)
**C5**	52.00 (1,041)	46.35 (928)	0.45 (9)	0.30 (6)	0.10 (2)	0.30 (6)	0.15 (3)	0.35 (7)	0 (0)	0 (0)	100 (2,002)
**C6**	49.48 (999)	49.63 (1,002)	0.15 (3)	0.15 (3)	0.15 (3)	0.05 (1)	0.05 (1)	0.25 (5)	0.05 (1)	0.05 (1)	100 (2,019)
**C7**	45.13 (917)	53.79 (1,093)	0.25 (5)	0.15 (3)	0.20 (4)	0 (0)	0.05 (1)	0.39 (8)	0.05 (1)	0.00 (0)	100 (2,032)
**Mean (%)/Total**	48.52 (6,839)	50.40 (7,027)	0.30 (40)	0.16 (22)	0.14 (19)	0.08 (10)	0.08 (11)	0.28 (38)	0.04 (5)	0.02 (3)	(14,014)
**Smokers**											
**S1**	48.03 (965)	50.67 (1,018)	0.40 (8)	0.10 (2)	0.20 (4)	0.14 (3)	0.10 (2)	0.25 (5)	0.05 (1)	0.05 (1)	100 (2,009)
**S2**	48.69 (985)	50.02 (1,012)	0.30 (6)	0.15 (3)	0.10 (2)	0.15 (3)	0.10 (2)	0.40 (8)	0 (0)	0.10 (2)	100 (2,023)
**S3**	49.36 (1,009)	49.46 (1,011)	0.44 (9)	0.10 (2)	0.10 (2)	0.10 (2)	0.15 (3)	0.29 (6)	0 (0)	0 (0)	100 (2,044)
**S4**	49.80 (1,011)	48.77 (990)	0.34 (7)	0.25 (5)	0.10 (2)	0.20 (4)	0.10 (2)	0.44 (9)	0 (0)	0 (0)	100 (2,030)
**S5**	47.10 (950)	51.02 (1,029)	0.55 (11)	0.10 (2)	0.20 (4)	0.20 (4)	0.15 (3)	0.69 (14)	0 (0)	0 (0)	100 (2,017)
**S6**	48.60 (990)	49.48 (1,008)	0.59 (12)	0.10 (2)	0.20 (4)	0.20 (4)	0.25 (5)	0.49 (10)	0.05 (1)	0.05 (1)	100 (2,037)
**S7**	47.04 (945)	51.42 (1,033)	0.50 (10)	0.20 (4)	0.10 (2)	0.10 (2)	0.15 (3)	0.45 (9)	0 (0)	0.05 (1)	100 (2,009)
**S8**	48.23 (969)	49.03 (985)	1.00 (20)	0.25 (5)	0.20 (4)	0.15 (3)	0.35 (7)	0.55 (11)	0.10 (2)	0.15 (3)	100 (2,009)
**S9**	49.63 (997)	47.98 (964)	0.90 (18)	0.10 (2)	0.15 (3)	0.15 (3)	0.20 (4)	0.75 (15)	0 (0)	0.15 (3)	100 (2,009)
**S10**	48.51 (975)	48.26 (970)	0.95 (19)	0.30 (6)	0.10 (2)	0.30 (6)	0.25 (5)	0.95 (19)	0.15 (3)	0.25 (5)	100 (2,010)
**Mean (%)/Total**	48.50 (9,796)	49.61 (10,020)	0.59 (120)	0.16 (33)	0.14 (29)	0.17 (34)	0.18 (36)	0.52 (106)	0.03 (7)	0.08 (16)	100 (20,197)

Statistical analyses were also applied to two subgroups of the smoking group: six individuals (identified as S2, S4, S5, S6, S7, and S8) who smoked more than 20 cigarettes per day and were considered heavy smokers, and four individuals (S1, S3, S9, and S10) who smoked less than 20 cigarettes per day and were classified as light smokers. There were no significant differences between the light and heavy smokers for either the total overall disomy frequency or for each of the chromosomes studied.

### Disomy of chromosomes 3, X, and Y

**Figure 2:**
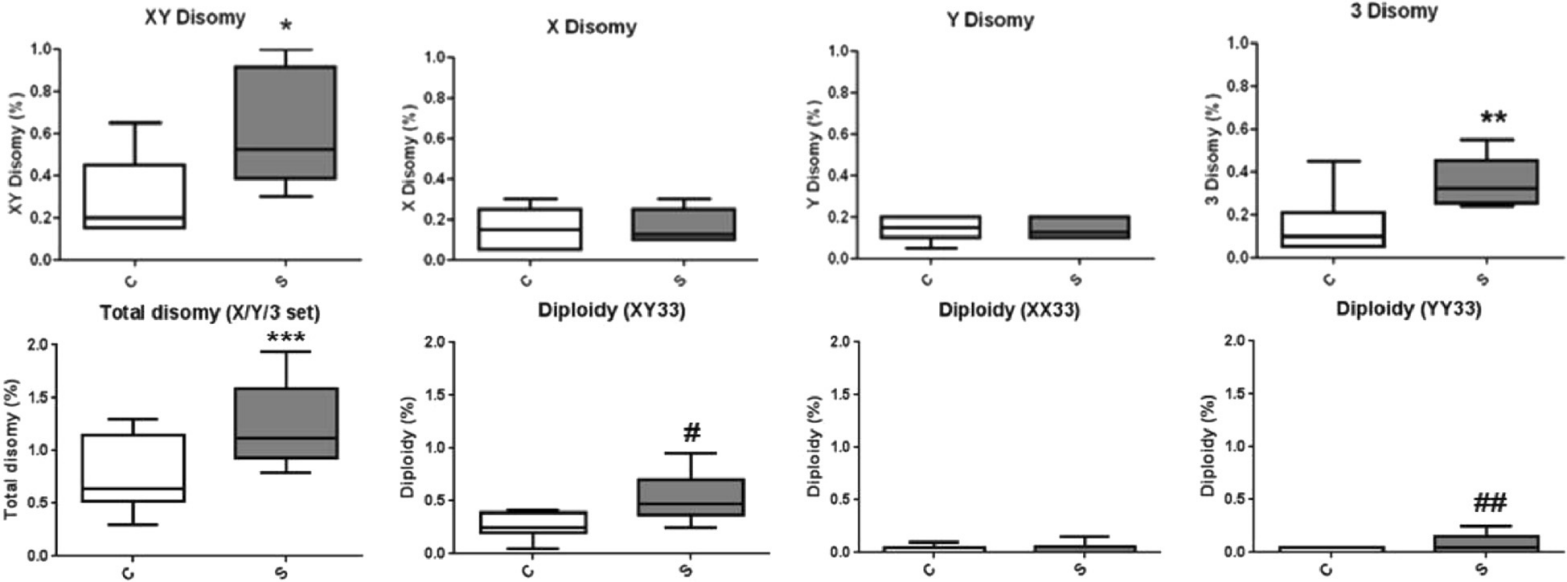
**Comparisons between the percentage of disomies and diploidies in sperm of controls (C) and smoking (S) groups.** (*) P < 0.0001, (**) P < 0.01. (***) P < 0.0001, (^#^) P < 0.0002 and (^##^) P < 0.0167.

Abnormalities leading to disomic sperm were present in both smoking and control groups. The control group had a mean of 0.73% ± 0.36 of total disomy for chromosomes 3, X, and Y, while the smoking group had a mean of 1.25% ± 0.41 (Figure 2), with a significant difference between the two groups (*P* < 0.0001).

The frequency of disomy 3 was significantly higher (*P* < 0.01), for the smoking group (0.35 ± 0.11) in comparison to the nonsmoker controls (0.15 ± 0.14). Analysis of the sex chromosomes also showed a significant increase (*P* < 0.0001) in the percentage of sperm carrying both sex chromosomes (XY disomy) in the smokers (0.59% ± 0.26), compared to nonsmoker controls (0.30% ± 0.19). Interestingly, when we compared the percentages of disomy for heavy smokers to those individuals classified as light smokers, we observed a slightly higher mean value for heavy smokers (0.67 ± 0.29) compared to the light smokers (0.54 ± 0.25). However, there was no statistical difference between these two subgroups. Other disomies involving sex chromosomes (XX and YY disomy) did not show any difference between smokers (0.16% ± 0.08 and 0.14 ± 0.05 for XX and YY disomy, respectively) and nonsmoker controls (0.16% ± 0.09 and 0.14% ± 0.05). Individual analysis of samples from both groups showed that XY disomy had the widest range in the smoking group, ranging from 0.3-1.0%. None of the individuals within this group were identified as outliers.

### Diploidy

The presence of disomy for all probes has been considered an indication that sperm may be diploid [14]. Using the set X/Y/3, it was possible to discriminate three types of diploid sperm: XY33, XX33, and YY33. The diploid sperm cells XY33 were found more frequently (*P* < 0.0002) in smokers (0.53% ± 0.21) than in nonsmoker controls (0.28% ± 0.13). The means of diploidy XX33 were 0.03% ± 0.05 in smokers and 0.04% ± 0.04 in controls, with no difference between chromosomes X or 3. However, for the diploid sperm YY33, the mean was 0.08% ± 0.08 in smokers and 0.02% ± 0.03 in controls, with a significant increase in the smoking group (*P* < 0.0167).

## Discussion

This study indicates that chromosome 3 may be associated with smoking-induced segregation anomalies. We observed a significant increase in total disomy of chromosomes 3, X, and Y in the smoking group in comparison to the control group. This finding was present in both smoking subgroups (light and heavy smokers), suggesting that the smoking habit, independent of the number of cigarettes used, is enough to generate meiotic errors. However, there is a wide set of definitions of light smoking, varying from individuals whom never smoked regularly to individuals that smoke 1–20 cigarettes per day [31,32]. Other authors have reported similar increases in disomy of chromosome 13 [33], disomy YY [34], disomy XX [35] and XY (reviewed [13]) associated with smoking. This present study is the first to describe similar impairment of chromosome 3 segregation in sperm derived from smokers.

The observed significant increase in the percentage of disomy of chromosome 3 suggests that this autosome may be unusually sensitive to the genotoxic damaging effects of tobacco in meiosis in males. Only one previous study has evaluated the segregation of chromosome 3, in a study unrelated to smoking [36]. Chromosome 3 presented at least three crossing-over regions and a larger synaptonemal complex, relative to the size reported for other chromosomes [37]. Those characteristics would be compatible with a meiotic segregation less prone to errors. However, Downie and colleagues [36] showed a high inter-individual variation in the frequency of disomy of chromosome 3. They also observed that the frequency of disomy 3 of 0.20% was more than three times higher than other autosomal chromosomes such as 7 (0.05%) and 16 (0.06%). This elevated frequency was also found in our study, with 0.15% of disomy 3 observed even in the control group. These preliminary data suggest that smoking may preferentially affect the meiotic segregation of chromosome 3, and imply that this chromosome is subject to more inter-individual variation in the population.

The exact mechanism to explain the association between smoking and increased aneuploidy in sperm is presently unknown. Some reports have shown an increase of oxidant substances and decreased antioxidant enzymes, providing a genotoxic environment for germ cells [38]. Smoking may also lead to deterioration of the sperm membrane, allowing greater interaction of extracellular substances with the nucleus [39]. Cotitine, a metabolite from nicotine, is able to cross the hematotestis barrier, and like other alkaloids, the drug can interact with the spindle [40]. The capacity of nicotine and others alkaloids to induce aneuploidy has also been confirmed in experimental systems [5,40].

We also identified a higher number of morphologically abnormal sperm cells in the smoking group than the controls (*P* < 0.036). We also observed seven smokers with asthenozoospermia and an apparent reduction in the total number of motile sperm, but we did not find any statistical differences when the smoking group was compared to controls for both progressive and total motility. Previously, Gaur and colleagues [41] verified that the motility was reduced even in light smokers compared to non-smokers. Also, they found a higher level of teratozoospermia in individuals who smoke more than 41 cigarettes per day. Our analysis confirmed that the smokers in our cohort had a reduction of the proportion of morphologically normal sperm that directly correlated with their fertilization success rate. Another study has described an inverse correlation between semen concentration and cell motility with sex chromosome disomies (XY disomy) [42]. Thus impairment of semen parameters and meiotic segregation anomalies could be due to both constitutive and exogenous factors. We excluded the oligozoospermic men based on the observations of Sarrate and colleagues [24] who evaluated chromosomal segregation in sperm of more than 300 infertile men and concluded that oligozoospermy was the only seminal parameter that correlated with the percentage of chromosome abnormalities.

The comparison of XY disomy between the two groups showed a significant increase in the smoking group. In contrast, the frequencies of disomies of XX and YY were not elevated in the smoking group. These findings suggest that pairing of particular homologues or specific events in the cell cycle are more prone to be disturbed by smoking. In this case, the segregation in meiosis I was more affected by tobacco consumption, rather than the separation of sister chromatids. Evaluating 11 smokers, Rubes and colleagues [34] verified an increase only in disomy YY, but not in XY or XX, while Robbins [35] observed an increase in disomy XX in smokers. In another study, no difference in sex chromosome disomies was identified between smokers and nonsmokers [33].

Our data suggest that errors in meiosis I in smokers are more frequent than in meiosis II. A possible explanation for this susceptibility in meiosis I in the sex chromosome pair could be the small number of chiasmas and the absence of a complete synaptonemal complex, two essential characteristics related to correct meiotic segregation [37,43]. Recently, McAuliffe and colleagues [42] also showed that a higher frequency of XY disomy was associated with low levels of concentration of sperm in ejaculated semen, while no difference was found in XX or YY disomies.

The frequency of diploid sperm cells with both sex chromosomes (identified as diploidy XY33) was higher in the smoking group. Rubes and colleagues [34] also identified an increase of diploid sperm cells in a smoking group, but their report did not specify the type of diploidy. We have observed that the frequency of XX33 and YY33 sperm was not higher in smokers than diploidy XY33, suggesting once more that meiosis I is more susceptible to some exogenous agent.

## Conclusion

In conclusion, our study has shown that chromosome 3 may be susceptible to smoking-related segregation anomalies. Our analysis using X/Y probes suggests that errors can occur in both meiosis I and II. Thus the overall elevated frequency of numerical chromosomal anomalies in the sperm of smokers compared to control fertile men is in keeping with the emerging literature of the potential damaging effects of tobacco toxins on chromosomal segregation mechanisms, and this could be associated with an elevated risk of infertility. Ideally male smokers should be strongly encouraged to cease smoking. We suggest that evaluative protocols for assisted reproductive technology could consider FISH analysis of sperm when the male partner continues to smoke.

## Methods

### Cohort

This study included two main groups. The control group was composed of seven fertile males in the age range of 31–38 years (33 ± 2.9). The smoking group included ten smokers in the age range of 23–48 years (33 ± 3.1). All males in both groups (controls and smokers) were fathers of at least one child in the last two years. None of non-smokers from the control group were ex-smokers and none of the controls or smokers had occupational exposure to pesticides or fungicides or had been submitted to hormone therapy or chemotherapy. Both groups include men with light alcohol consumption (1–4 drinks per month) except for one smoking individual, identified as S10, who was defined as an abstainer (less than 12 drinks per year).

### Ethical approval

The smoking and control groups were informed about the study, and all the patients signed the consent form approved by the Hospital Ethics Committee of Ribeirão Preto Medical School, University of Sao Paulo (process HCRP 6092/2008).

### Karyotype

A peripheral blood culture of all men from both groups was performed according to standard cytogenetic methods. Chromosomal analysis by GTG banding was carried out in 100 metaphases for mosaicism exclusion.

### Spermogram

Each sperm sample was collected in a sterile collector and an aliquot was used for spermogram analysis and FISH. Seminal parameters including concentration, morphology, motility, and vitality of sperm were analyzed accordingly to World Health Organization (WHO) [44]. All controls used in this study had seminal parameters that were in keeping with the reference limits suggested by WHO [44]. The parameters considered for the controls were 39 (range 33–46) ×10^6^ per ejaculate for total sperm number, 15 (12–16) ×10^6^ per ml for sperm concentration, 40% (38–42%) for total motility (Progressive = PR; Non-progressive = NP), 58% (55–63%) for vitality (% of live spermatozoa) and 4% (3.0–4.0%) for sperm morphology (% of normal forms).

### Fluorescence in situ hybridization in sperm

Sperm decondensation and hybridization procedures were performed as described by Juchniuk de Vozzi et al. [45]. The slides were made with 5 μl of the sample and stored at −20°C.

Multicolor interphase FISH studies of sperm preparations were performed using a probe set for each sample: a triple-color FISH with specific probes for chromosomes X (CEP X, Locus DXZ1), Y (SE Y class q arm, Yqh), and 3 (SE 3, Locus D3Z1 and SE 3, Locus D3Z) from Kreatech Diagnostics, USA. Two chromosome 3 probes were combined to generate a yellow color. Hybridization efficiency was 97.5%, ranging between 97% and 98%.

### Sperm disomy and diploidy scoring

The FISH slides were coded and enumerated by two independent analysts using an Olympus BX-40 microscope (Olympus, UK) and Applied Imaging software (Applied Imaging, UK).

Scoring criteria were based on Downie and colleagues [14]. The selected sperm cells did not exhibit overlaps and were symmetrically oval with normal tails. Scored signals presented an equal size and the same fluorescence intensity and were clearly located within the nucleus. The sperm classified as disomic showed two signals of similar size, and both were separated by at least half a signal domain. At least 2,000 sperm cells were analyzed per individual.

### Statistical analysis

The data was analyzed by Statistical Analysis System software (SAS) using GENMOD which fits a generalized linear model to the data by a maximum likelihood estimation, considering the analyzed frequencies distribution. To compare controls and smokers, the analysis was performed using a two-parameter gamma test for all phenotypic variables related to sperm (motility, concentration, vitality, and morphology), which followed a continuous probability distribution. Poisson distribution was considered to analyze the frequencies of disomies and diploidies in sperm because these discontinuous variables were not normally distributed and occurred as independent events. All data are shown as mean ± SD.
